# Tobacco Education in Doctor of Pharmacy Programs in the United States (2021–2022)

**DOI:** 10.1016/j.ajpe.2023.100120

**Published:** 2023-05-14

**Authors:** Karen Suchanek Hudmon, Alexa M. Lahey, Julia S. Czarnik, Nervana Elkhadragy, Robin L. Corelli

**Affiliations:** a Purdue University, College of Pharmacy, West Lafayette, IN, USA; b University of Wyoming, School of Pharmacy, Laramie, WY, USA; c University of California San Francisco, School of Pharmacy, San Francisco, CA, USA

**Keywords:** Tobacco cessation, Smoking cessation, Pharmacy, Education, Curriculum

## Abstract

**Objective:**

To characterize (1) tobacco cessation content, delivery, and assessment methods, (2) faculty perceptions of content adequacy, and (3) faculty interest in enhancing curricular content as a result of pharmacists’ new, expanding role in prescribing tobacco cessation medications.

**Methods:**

One faculty member responsible for teaching tobacco cessation-related content at each college and school of pharmacy was invited to participate in a national, web-based survey. Survey items assessed various aspects of tobacco education and gauged faculty interest in attending a train-the-trainer program and integrating Tobacco Treatment Specialist training as part of the curriculum at their institution.

**Results:**

Of 132 survey respondents (93.0% response), 98.5% reported integrating tobacco cessation into the required curriculum, and 15.2% integrated the content into an elective course. The median number of formal educational hours was 5.0 (range, 1.0–18.0). One-third (33.3%) assessed students’ tobacco cessation competency using objective structured clinical examinations. Most (83.8%) felt that their institution has adequate faculty expertise to teach comprehensive tobacco cessation, and 98.5% were interested in attending a train-the-trainer program for pharmacy faculty to learn to educate students on the latest developments of pharmacist-provided tobacco cessation. Similarly, 95.4% were interested in incorporating Tobacco Treatment Specialist training into their Doctor of Pharmacy curriculum.

**Conclusion:**

Given the expanding scope of pharmacists’ practice for prescribing tobacco cessation medications, there is a need to enhance curricular content in Doctor of Pharmacy programs. Current faculty expressed interest in expanding coursework to enable their graduates to work at the top of their license when treating tobacco use and dependence.

## 1. Introduction

Tobacco use is the leading known preventable cause of morbidity and mortality in the United States (US), resulting in more than 480,000 deaths and costing $288.9 billion annually in direct medical care and lost productivity from premature mortality each year.^1^ Although substantial efforts have led to a reduced prevalence of tobacco use, in 2021, an estimated 46.0 million US adults (18.7%) were current tobacco users.^2^ There remains a significant need for healthcare providers to become more engaged in tobacco cessation counseling, which is among the most effective methods of intervention.^3–7^ Pharmacists, as one of the most accessible healthcare providers in community settings, are uniquely positioned to provide tobacco cessation assistance and medications for patients who are ready to quit. In recent years, the scope of practice for pharmacists has expanded to include the ability to prescribe cessation medications. As of March 2023, autonomous prescribing has been implemented or is in the process of being implemented in 17 states, with 9 of these states including all US Food and Drug Administration-approved medications for cessation.^8^

Research has demonstrated that healthcare professionals who receive specialized training for treating tobacco dependence are more likely to incorporate cessation interventions in clinical practice^9^. However, over the past 2 decades, the pharmacy profession has been the only health discipline to systematically and sustainably incorporate comprehensive tobacco cessation training into its core curricula for students.^10–14^ This is, in large part, due to the development and dissemination of a shared, comprehensive tobacco cessation training program for health professional students (Rx for Change: Clinician-Assisted Tobacco Cessation program; https://rxforchange.ucsf.edu)^15^ that has witnessed widespread and sustained use over more than 2 decades.^14,16–18^ As part of a nationwide project, in 2003–2005, faculty members (*n* = 191) representing 89 of 91 US colleges and schools of pharmacy (97.8%) participated in a 2.5-day train-the-trainer program to learn to teach the Rx for Change curricular content.^13^ In 2016, more than a decade later, a national survey conducted by the American Association of Colleges of Pharmacy^14^ estimated that 73.5% were currently using the Rx for Change program materials in their Doctor of Pharmacy (PharmD) curricula, providing evidence for the longevity of a shared curriculum approach. However, due to the large increase in the number of colleges and schools of pharmacy over the past 25 years, along with pharmacists’ expanding role in prescribing tobacco cessation medications,^19,20^ new data are needed to enhance our understanding of the current environment surrounding tobacco cessation education in PharmD curricula.

This study aimed to conduct a national survey of faculty members in colleges and schools of pharmacy to characterize (1) current tobacco cessation content, delivery methods, and assessment methods, (2) faculty perceptions of tobacco cessation content, and (3) faculty interest and perceived need to modify or enhance curricular content as a result of pharmacists’ expanding roles in prescribing tobacco cessation medications. These data will provide guidance for future efforts to prepare the pharmacy profession for its evolving role in tobacco cessation.

## 2. Methods

For each of 142 accredited, US-based PharmD programs, faculty members eligible for participation in this study (ie, instructors responsible for teaching tobacco cessation-related content in the curriculum) were identified through school websites, through personal contacts/colleagues, or by contacting department chairs or heads. A recruitment email was sent to each contact, requesting survey completion if they were the primary tobacco instructor. If the targeted faculty member was (1) not the most appropriate person to complete the survey or (2) unable to participate, they were asked to identify an appropriate colleague at their institution.

Survey procedures included 4 email communication attempts: 1 recruitment invitation with the consent document and the survey, 3 reminders, and a maximum of 2 follow-up telephone calls. Participants who completed the 10-minute survey received a $30 Amazon.com gift card as compensation for their time. All study procedures were approved by the Human Research Protection Program at Purdue University. Surveys were conducted using Qualtrics during the Spring and Summer of 2022.

Modeled after prior published surveys of tobacco cessation curricular content,^21–23^ study measures included school characteristics, tobacco cessation content taught, and content delivery and assessment methods. Additionally, interest in (1) attending a future train-the-trainer program and (2) integrating a more comprehensive level of content into their curriculum was assessed.

Pharmacy schools were characterized based on the number of students entering the PharmD program in the 2021–2022 school year. A comprehensive list of schools, along with public vs private status, was retrieved from the American Association of Colleges of Pharmacy databases.^24^

Incorporation of tobacco cessation content was assessed for required courses, elective courses devoted entirely to tobacco cessation, and other elective courses that included tobacco content. Participants reported the total number of hours of required curricular time dedicated to tobacco-related content over the 2021–2022 academic year, and in which professional year(s) the content was taught.

Respondents were asked if the Rx for Change program was currently being used to teach tobacco cessation as part of required coursework. If yes, they were asked to specify which components were being used: (1) PowerPoint lecture slides, (2) case scenarios for role-playing, (3) ancillary student handouts, (4) video of the Surgeon General’s welcome message, (5) videos of tobacco cessation counseling sessions, (6) tobacco trigger tape video vignettes, (7) hands-on skills training with placebo or samples of nicotine replacement therapy formulations, (8) tobacco-specific virtual patients, (9) or tobacco-specific standardized patients/objective standardized patients/Objective Structured Clinical Examination (OSCE) cases.

Faculty were also asked whether specific topic areas were taught in the required coursework: (1) epidemiology of tobacco use, (2) forms of tobacco and e-cigarettes/vaping, (3) pharmacology of nicotine and principles of addiction, (4) drug interactions with tobacco smoke, (5) comprehensive counseling interventions (the 5 A’s [ask, advise, assess, assist, arrange]), (6) brief counseling interventions (the Ask-Advise-Refer model), and (7) medications for quitting. For each of the topic areas indicated as being included in required coursework, faculty were then asked if topics were inadequately covered, adequately covered, or excessively covered in the required (core) didactic curriculum.

Educational methods used to deliver tobacco-focused content in required coursework were assessed. Non-mutually exclusive response options included (1) classroom lectures, (2) home study podcasted lectures or webinars, (3) provision of required reading materials, (4) case study discussion, (5) Rx for Change Virtual Patients for tobacco cessation, (6) case-based counseling/student role-playing activities, and (7) case-based counseling/standardized patient encounters with formal feedback.

Faculty were asked to specify which assessment method(s) were being used to evaluate their students’ competency for tobacco cessation counseling. Response options included multiple-choice examination, short answer examination, case-based multiple-choice examination, case-based short answer examination, oral examination, and OSCE. Responses were not mutually exclusive.

Participants were asked if they felt that their school had adequate faculty expertise to teach comprehensive tobacco cessation; response options were yes, no, and I am not sure. Additionally, survey items assessed their interest in (1) attending a train-the-trainer program to learn to teach the latest developments in pharmacist-provided tobacco cessation (with travel provided at no cost) and (2) incorporating Tobacco Treatment Specialist (TTS) training into their pharmacy curriculum if guidance and resources were provided at no cost. A link to the Association for the Treatment of Tobacco Use and Dependence (ATTUD) website, which provides a description of the TTS role and multi-day training, was provided.^25^ According to ATTUD, a TTS is “a professional who possesses the skills, knowledge, and training to provide effective, evidence-based interventions for tobacco dependence across a range of intensities.”^25^ They also engage in educating others to assist patients with quitting. Response options for this item were interested, interested but I need more information, and not interested.

Descriptive statistics were computed to summarize the responses. Statistics were run using IBM SPSS version 28.0.

## 3. Results

Of 142 schools of pharmacy, the research survey was completed by 132 (93.0%). Of these, 67 (50.8%) were public institutions and 65 (49.2%) were private, with a median 2021–2022 entering class size of 78.5 (range, 14–310). All but one of the 46 states with 1 or more pharmacy schools were represented in the respondent population.

Of all respondents, 98.5% and 15.2% indicated that tobacco education and intervention skills were integrated into required coursework and elective coursework, respectively. Four schools (3.0%) offered an elective course dedicated entirely to tobacco cessation education and intervention. Across the 2021–2022 academic year, the median total number of hours of tobacco education in required coursework was 5.0 h (range, 1–18), with 39.2% of schools achieving the recommended minimum of 6.0 h.^26^ Just over half (55.5%) included content in the first professional year, 57.4% in the second year, and 31.5% in the third year.

More than half of respondents (*n* = 76; 57.6%) indicated that content from Rx for Change was currently being used to teach tobacco cessation as part of required coursework at their institution; of these, the majority reported using ancillary student handouts (90.8%), PowerPoint lecture slides (89.3%), and case scenarios for role-playing (54.8%). Other Rx for Change program components reported being used to teach tobacco cessation included hands-on skills training with placebo or samples of nicotine replacement therapy formulations (40.5%), videos of tobacco cessation counseling sessions (37.0%), tobacco-specific virtual patients (24.3%), tobacco trigger tape video vignettes (23.3%), tobacco-specific standardized patients/objective standardized patients/OSCE cases (17.8%), and video of the Surgeon General’s welcome message (13.7%).

When asked to report which tobacco-related content areas were addressed in the core curriculum, and if taught, whether they perceived the content to be inadequately, adequately, or excessively covered (Table 1), the most commonly addressed topics were medications for quitting (100%), comprehensive counseling interventions (94.7%), and nicotine pharmacology and principles of addiction (93.2%). Forms of tobacco and e-cigarettes/vaping were the topic most commonly perceived to be inadequately covered, by 29.2% of faculty respondents.

Educational methods used to deliver tobacco content in required coursework included (1) classroom lectures (95.4%), (2) case study discussion (86.0%), (3) provision of required reading materials (65.1%), (4) case-based counseling/student role-playing activities (64.3%), (5) case-based counseling/standardized patient encounters with formal feedback (38.4%), (6) home study podcasted lectures or webinars (23.4%), and (7) virtual patients for tobacco cessation (16.7%).

Assessment method(s) used to evaluate students’ competency for tobacco cessation counseling included multiple-choice examination (100%), case-based multiple-choice examination (82.9%), OSCE (33.3%), case-based short answer examination (30.5%), short answer examination (25.2%), and oral examination (6.8%).

Most respondents felt that their faculty had adequate expertise to teach comprehensive tobacco cessation (*n* = 109; 83.8%); 15 (11.5%) were not sure, and 6 (4.6%) did not believe there was adequate faculty expertise. More than two-thirds (*n* = 90; 68.2%) indicated that they were interested in attending a train-the-trainer program to learn to teach the latest developments in pharmacist-provided tobacco cessation ; 40 (30.3%) were interested but needed more information, and 2 (1.5%) were not interested. When asked regarding interest in incorporating TTS training into their pharmacy curriculum, 76 (58.0%) were interested, 49 (37.4%) were interested but needed more information, and 6 (4.6%) were not interested.

## 4. Discussion

As the role of pharmacists in treating tobacco use and dependence continues to evolve, PharmD graduates must be adequately prepared to engage with patients who are interested in quitting. Although all pharmacists are trained to dispense and provide counseling for medications prescribed by other providers, it is now increasingly important that pharmacists are also able to effectively evaluate the appropriateness and need, select, and prescribe these medications. Furthermore, many state protocols for prescribing require that pharmacists be capable of conducting follow-ups with recent quitters,^20^ which involves reassessment of patients’ treatment regimens and making modifications, as needed, to ensure safety and efficacy. Historically, content in PharmD curricula has not addressed the prescribing aspects of tobacco treatment.^21^

In light of these major changes in scope of practice, approaches to teaching and assessment must ensure that key competencies are achieved. In terms of the overall tobacco content in PharmD curricula nationwide, there is room for improvement—survey results revealed a median of 5.0 h of tobacco content being taught, which is below the 6.0 h recommended minimum established in 2013,^26^ when only 1 state (New Mexico)^27^ had prescriptive authority for cessation medications. Increasing the amount of in-class instruction is always challenging in an already-impacted curriculum; however, there are strategies to circumvent this, including pre-class assignments (web-based modules, readings) with quizzes to ensure knowledge mastery before in-class lectures, which then can focus on active learning and case-based discussions. Faculty respondents indicated a wide range of teaching and learning tools, with more than half using materials from the Rx for Change shared tobacco curriculum. The Rx for Change content is updated annually, which conserves valuable faculty resources and has witnessed widespread, sustained use for more than 2 decades.^14,16,18^

Results of this study provide insight regarding assessment methods for tobacco cessation knowledge and associated competencies, with 38.4% implementing case-based counseling/standardized patient encounters with formal feedback and 33.3% implementing tobacco cessation OSCE cases for assessment. A limitation of our survey is that it did not obtain granular details regarding the number of OSCE cases or characteristics of standardized patient roles. Ideally, a tobacco OSCE will include 3 cases, with each patient being in a different stage of quitting: not ready to quit, ready to quit, and recent quitter. In states where pharmacists can prescribe cessation medications, it is advisable to build the cases around the logistics of the state’s protocol. For example, in Indiana, North Dakota, and Vermont (which have nearly identical protocols),^20^ 1 case would involve a patient who is not ready to quit, a second case would require the student to conduct an intake assessment and develop a treatment plan for a patient who is ready to quit, and a third case would involve a 14-day follow-up with a recent quitter, to reassess adequacy of the treatment regimen and discuss any challenges that the patient is having with quitting. When a 3-station OSCE is not feasible, educators can integrate tobacco use into other OSCE cases addressing tobacco-related diseases (eg, asthma, cancer, cardiovascular disease, diabetes, pulmonary disease, etc.). Several tobacco-specific standardized patient cases, along with an OSCE tutorial and other tools, are accessible to faculty on the Rx for Change website, at https://rxforchange.ucsf.edu.^15^

Most respondents reported that their institution had adequate faculty expertise to teach comprehensive tobacco cessation; however, 6 did not believe there was adequate expertise. Of these, 5 programs were not in existence to benefit from the tobacco cessation train-the-trainer programs that were provided in 2003, 2004, and 2005.^13^ Despite confidence in faculty expertise among the vast majority of PharmD programs, more than 95% of respondents expressed interest in (1) attending a train-the-trainer program for faculty to learn the latest developments for treating tobacco use and dependence, and (2) integrating a higher level of training for PharmD students, ie, synonymous with the 4-day training that is provided for TTS certifications. This interest, along with wide ranges in the number of hours taught, content covered, and methods of delivery and assessment, suggests that the profession could benefit from another national training initiative for pharmacy faculty. This initiative could include development and dissemination of shared teaching and learning materials (eg, flipped classroom approaches, OSCEs) that move beyond the standard content currently taught in PharmD curricula. Challenges will include aligning curricular priorities and identifying creative approaches for integrating this new content into already-impacted coursework. Finally, nearly 2 decades have elapsed since the 2003–2005 Rx for Change faculty training initiatives, and the colleges and schools of pharmacy would benefit from train-the-trainer programs for the newer faculty members who are now teaching tobacco cessation.

Strengths of this study include the robust response rate and ability to identify content areas to address in future training efforts. Limitations include the self-reported nature of the data and the corresponding lack of validity assessments. Additionally, although the brevity of the survey (along with a financial incentive for completion) likely enhanced our response rate, this also limited our ability to obtain more granular data pertaining to faculty perceptions and implementation strategies. Future research should attempt to characterize these important faculty experiences more carefully.

## 5. Conclusion

Given the expanded scope of pharmacists’ practice for prescribing tobacco cessation medications, there is substantial interest in modifying curricular content in US colleges and schools of pharmacy. Faculty express interest in expanding coursework, including more extensive coverage of e-cigarettes, vaping, and other forms of tobacco. Broader education on new developments in current tobacco treatment approaches will help ensure that graduates are capable of working at the top of their license when treating tobacco use and dependence.

## Figures and Tables

**Table 1:** Tobacco-Related Topics Addressed in Core Curriculum and Respondents’ Perceived Adequacy of Coverage (*n* = 132).

Topic	n (%)^a^	Perceived adequacy^b^		
	
		Inadequately covered	Adequately covered	Excessively covered

Epidemiology	115 (81.7)	20 (17.4)	92 (80.0)	3 (2.6)
Forms of tobacco and e-cigarettes/vaping	107 (81.1)	31 (29.2)	74 (69.8)	1 (0.9)
Nicotine pharmacology and principles of addiction	123 (93.2)	14 (11.6)	104 (86.0)	3 (2.5)
Drug interactions with tobacco smoke	108 (81.8)	15 (14.2)	92 (85.8)	0
Comprehensive counseling interventions (the 5A’s^c^)	125 (94.7)	19 (15.4)	92 (74.8)	12 (9.8)
Brief counseling interventions (Ask-Advise-Refer)	100 (75.8)	16 (16.3)	80 (81.6)	2 (2.0)
Medications for quitting	132 (100.0)	5 (3.8)	117 (90.0)	8 (6.2)

a Number of respondents reporting that the topic was addressed in their core curriculum.

b Not all schools addressing each topic responded to the corresponding “perceived adequacy” survey item.

c Ask about tobacco use, Advise patients to quit, Assess readiness to quit, Assist with quitting, and Arrange follow-up.

## References

[1] United States Department of Health and Human Services. The health consequences of smoking—50 years of progress. A report of the surgeon general. United States Department of Health and Human Services, Centers for Disease Control and Prevention, National Center for Chronic Disease Prevention and Health Promotion, Office on Smoking and Health, Atlanta, GA; 2014.

[2] Cornelius ME, Loretan CG, Jamal A, Tobacco product use among adults—United States, 2021. MMWR Morb Mortal Wkly Rep. 2023;72(18):475–483. 10.15585/mmwr.mm7218a1 37141154 PMC10168602

[3] Stead LF, Buitrago D, Preciado N, Physician advice for smoking cessation. Cochrane Database Syst Rev. 2013;2013(5):CD000165. 10.1002/14651858.CD000165.pub4 23728631 PMC7064045

[4] Rice VH, Heath L, Livingstone-Banks J, Hartmann-Boyce J. Nursing interventions for smoking cessation. Cochrane Database Syst Rev. 2017;12(12):CD001188. 10.1002/14651858.CD001188.pub5 29243221 PMC6486227

[5] Carson-Chahhoud KV, Livingstone-Banks J, Sharrad KJ, Community pharmacy personnel interventions for smoking cessation. Cochrane Database Syst Rev. 2019;2019(10):CD003698. 10.1002/14651858.CD003698.pub3 31684695 PMC6822095

[6] Holliday R, Hong B, McColl E, Livingstone-Banks J, Preshaw PM. Interventions for tobacco cessation delivered by dental professionals. Cochrane Database Syst Rev. 2021;2(2):CD005084. 10.1002/14651858.CD005084.pub4 33605440 PMC8095016

[7] Fiore MC, Jaén CR, Baker TB, Treating tobacco use and dependence. Update. Clinical Practice Guideline. Rockville, MD: United States Department of Health and Human Services, Public Health Service; 2008.

[8] National Alliance of State Pharmacy Associations. Pharmacist prescribing: tobacco cessation Aids. National Alliance of State Pharmacy Associations. https://naspa.us/resource/tobacco-cessation/. Accessed May 11, 2023.

[9] Carson KV, Verbiest ME, Crone MR, Training health professionals in smoking cessation. Cochrane Database Syst Rev. 2012;2012(5):CD000214. 10.1002/14651858.CD000214.pub2 22592671 PMC10088066

[10] Hudmon KS, Kroon LA, Corelli RL, Training future pharmacists at a minority educational institution: evaluation of the Rx for change tobacco cessation training program. Cancer Epidemiol Biomarkers Prev. 2004;13(3):477–481. 10.1158/1055-9965.477.13.3 15006926

[11] Hudmon KS, Corelli RL, Chung E, Development and implementation of a tobacco cessation training program for students in the health professions. J Cancer Educ. 2003;18(3):142–149. 10.1207/S15430154JCE1803_07 14512261

[12] Corelli RL, Kroon LA, Chung EP, Statewide evaluation of a tobacco cessation curriculum for pharmacy students. Prev Med. 2005;40(6):888–895. 10.1016/j.ypmed.2004.10.003 15850892

[13] Corelli RL, Fenlon CM, Kroon LA, Prokhorov AV, Hudmon KS. Evaluation of a train-the-trainer program for tobacco cessation. Am J Pharm Educ. 2007;71(6):109. 10.5688/aj7106109 19503693 PMC2690925

[14] Lang W, Elkhadragy N, Hudmon KS. Not Blowing Smoke: academic pharmacy aims to reduce tobacco use to ashes. Acad Pharm Now. 2016;9(2):12–20.

[15] University of California San Fransisco. Rx for Change: Clinician-Assisted Tobacco Cessation. University of California San Francisco, San Francisco, CA; 1999–2023. https://rxforchange.ucsf.edu. Accessed May 11, 2023.

[16] Elkhadragy N, Corelli RL, Zillich AJ, Campbell NL, Hudmon KS. Long-term evaluation of a train-the-trainer workshop for pharmacy faculty using the RE-AIM framework. Res Social Adm Pharm. 2021;17(9):1562–1569. 10.1016/j.sapharm.2020.11.018 33551208 PMC8286451

[17] Elkhadragy N, Corelli RL, Russ AL, Faculty perceptions of a tobacco cessation train-the-trainer workshop and experiences with implementation: A qualitative follow-up study. Res Social Adm Pharm. 2019;15(12):1436–1445. 10.1016/j.sapharm.2019.01.005 30737194 PMC6616006

[18] Elkhadragy N, Aviado J, Huang H, Corelli RL, Hudmon KS. Shared tobacco cessation curriculum website for health professionals: longitudinal analysis of user and utilization data over a period of 15 years. JMIR Med Educ. 2021;7(2):e20704 10.2196/20704 34032582 PMC8188318

[19] Adams AJ, Hudmon KS. Pharmacist prescriptive authority for smoking cessation medications in the United States. J Am Pharm Assoc. 2018;58(3):253–257. 10.1016/j.japh.2017.12.015 PMC9363931 29426612

[20] Hilts KE, Corelli RL, Vernon VP, Hudmon KS. Update and recommendations: pharmacists’ prescriptive authority for tobacco cessation medications in the United States. J Am Pharm Assoc. 2022;62(5):1531–1537. 10.1016/j.japh.2022.06.005 PMC9464677 35953378

[21] Hudmon KS, Bardel K, Kroon LA, Fenlon CM, Corelli RL. Tobacco education in U.S. schools of pharmacy. Nicotine Tob Res. 2005;7(2):225–232. 10.1080/14622200500055392 16036279

[22] Houston LN, Warner M, Corelli RL, Fenlon CM, Hudmon KS. Tobacco education in US physician assistant programs. J Cancer Educ. 2009;24(2):107–113. 10.1080/08858190902854475 19431026

[23] Hudmon KS, Mark M, Livin AL, Corelli RL, Schroeder SA. Tobacco education in U.S. respiratory care programs. Nicotine Tob Res. 2014;16(10):1394–1398. 10.1093/ntr/ntu113 25031314 PMC4207876

[24] Accreditation Council for Pharmacy Education. Programs by status. Accreditation Council for Pharmacy Education https://www.acpe-accredit.org/accredited-programs-by-status/. Accessed May 11, 2023.

[25] Association for the Treatment of Tobacco Use and Dependence (ATTUD). Core competencies for evidence-based treatment of tobacco Dependence. ATTUD 2005. https://www.attud.org/assets/docs/Standards.pdf. Accessed May 11, 2023.

[26] McBane SE, Corelli RL, Albano CB, The role of academic pharmacy in tobacco cessation and control. Am J Pharm Educ. 2013;77(5):93. 10.5688/ajpe77593 23788804 PMC3687126

[27] New Mexico Regulation and Licensing Department. Protocol for pharmacist prescribing for tobacco cessation. New Mexico Regulation and Licensing Department. https://www.nmpharmacy.org/Resources/Documents/rx%20authority/protocol-tobacco.pdf. Accessed May 11, 2023.

